# Distributional bias compromises leave-one-out cross-validation

**Published:** 2024-06-03

**Authors:** George I. Austin, Itsik Pe’er, Tal Korem

**Affiliations:** 1 Department of Biomedical Informatics, Columbia University Irving Medical Center, New York, NY, USA; 2 Program for Mathematical Genomics, Department of Systems Biology, Columbia University Irving Medical Center, New York, NY, USA; 3 Department of Computer Science, Columbia University, New York, NY, USA; 4 Department of Obstetrics and Gynecology, Columbia University Irving Medical Center, New York, NY, USA

## Abstract

Cross-validation is a common method for estimating the predictive performance of machine learning models. In a data-scarce regime, where one typically wishes to maximize the number of instances used for training the model, an approach called ‘leave-one-out cross-validation’ is often used. In this design, a separate model is built for predicting each data instance after training on all other instances. Since this results in a single test data point available per model trained, predictions are aggregated across the entire dataset to calculate common rank-based performance metrics such as the area under the receiver operating characteristic or precision-recall curves. In this work, we demonstrate that this approach creates a negative correlation between the average label of each training fold and the label of its corresponding test instance, a phenomenon that we term distributional bias. As machine learning models tend to regress to the mean of their training data, this distributional bias tends to negatively impact performance evaluation and hyperparameter optimization. We show that this effect generalizes to leave-P-out cross-validation and persists across a wide range of modeling and evaluation approaches, and that it can lead to a bias against stronger regularization. To address this, we propose a generalizable rebalanced cross-validation approach that corrects for distributional bias. We demonstrate that our approach improves cross-validation performance evaluation in synthetic simulations and in several published leave-one-out analyses.

## Introduction

One of the most important challenges of devising any machine learning model, whether it is as simple as a linear model over structured data or as complex as a large language model applied to free text, is to robustly evaluate its performance^1–9^. A common strategy for such evaluations is “cross-validation”^10^. In cross-validation, the data is divided into parts (“folds”, e.g., a tenth of the data), which are then each used as a held-out test set for a model trained on the rest of the dataset. The predictions made by the model on each held-out test set are then compared to the hidden labels, either separately for each fold or in aggregate across folds (Fig. S1). In the common use-case of binary classification, performance is typically assessed via metrics such as the area under the receiver operator characteristic curve^11^ (auROC) or precision-recall curve^12^ (auPR), which are determined by the rank order of the certainty of the model’s predictions^10^.

Training and evaluating models in data-scarce scenarios in which the acquisition of each data instance is challenging or expensive is difficult. In datasets that are small to begin with, reducing the data further by holding out multiple points as a test set makes it significantly harder for the model to infer the underlying signal, reducing performance. To keep nearly all samples available for training each model, a common variation of this approach, known as ‘leave-one-out cross-validation^13^ (LOOCV), treats every individual point as an independent test set, thus maximizing the number of training samples used for every model trained. LOOCV can also be generalized for keeping any number of samples as a test fold, known as “leave-P-out cross-validation”^14–16^ (LPOCV), where P is the number of samples left-out per test fold.

In this work, we demonstrate that standard implementations of LPOCV introduce distributional bias – a negative correlation between the average label of the corresponding training and test sets – and show that it negatively affects performance evaluation. We demonstrate this phenomenon via a simple predictor which takes advantage of this bias to obtain perfect scores on any LOOCV task. In practice, however, due to the tendency of machine learning models to regress to the mean of the training set labels, this bias tends to decrease rank-based performance evaluation metrics, such as auROC or auPR. We show that this impacts not just performance evaluation, but also hyperparameter optimization, with distributional bias favoring the selection of models with weaker regularization. To address this, we propose a generalizable correction for LOOCV, called “Rebalanced LOOCV”, (RLOOCV), in which a sample with the opposite label to the held-out test point is removed from each training set. We demonstrate that this correction consistently increases measured machine learning performances in published LOOCV-based evaluation of models predicting preterm birth, chronic fatigue syndrome, and adverse events for immune checkpoint inhibitor treatment.

## Results

### Distributional bias leaks information on test labels in LOOCV and LPOCV

**Figure 1 |:**
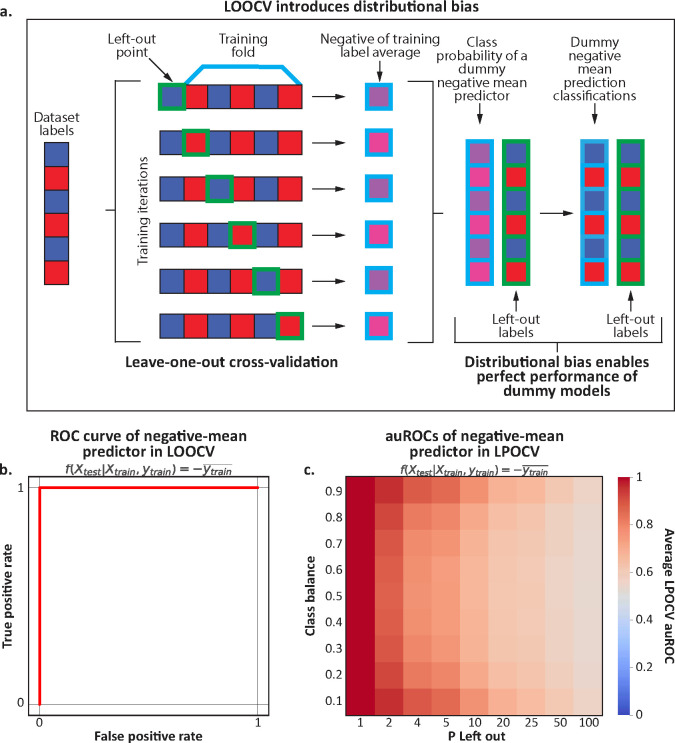
Distributional bias leaks the test set label in leave-one-out cross-validation. **a,** An illustration of how distributional bias occurs in LOOCV. When a held-out point belongs to either class, the class average of the remaining dataset shifts slightly in the other direction. As a result, a dummy predictor that returns the negative of the average training class label would produce predictions that are perfectly correlated with the actual labels. **b,** Receiver operator characteristic (ROC) curve for this dummy negative-mean predictor. The auROC is 1 under any scenario regardless of the underlying data. **c,** A heatmap showing the average auROC of the same dummy negative-mean predictor under different class balances and P-left-out schema on randomly generated labels, with resulting auROCs consistently over the expected null auROC of 0.5.

In a classification task evaluated using LOOCV, every removal of a sample as a test set changes the sample mean of the training set labels. Across test sets, these shifts in label distribution create a perfect negative correlation (r=−1) between the means of training set labels and the labels of the test sets (Fig. 1a). In this scenario, information about the test label “leaks” into the training set: the held-out labels can be uniquely inferred by simply observing the training label average. To demonstrate this leakage, we simulated random data and showed that a dummy model that only predicts the negative mean of the training set’s labels achieves perfect auROC and auPR of 1 (Figs. 1b, S2a; Methods). This is regardless of the underlying data, and in a manner completely independent from the test data and labels.

Next, we sought to evaluate whether this effect generalizes to more general LPOCV scenarios. To do so, we simulated random datasets of 250–300 samples while varying the class balance (from 10% to 90%) and the number of samples left out per fold (from 1 to 100; Methods). Evaluating the same dummy model on this data, we observed that the impact of distributional bias decreases as the fold size increases, starting with auROCs of 1.00 at N=1 and decreasing to an average auROC of 0.55 (range of 0.54–0.57) at N=100 (Fig. 1c). However, even at N=100, the dummy predictor still obtained significantly higher auROCs than a random guess (one-sided t-test p<0.001), implying that distributional bias is still an issue even in this setting. We further observed an interaction between the class balance, held-out P size, and the impact of distributional bias, with higher impact on more extreme class balances. For example, the dummy model for P=4 had mean±std auROCs of 0.88±0.03 and 0.88±0.03 for class balances of 10% and 90%, respectively, while the same model had a mean±std auROCs of 0.76±0.04 for the same P at a class balance of 50% (Figs. 1c, S2b). Complementarily, a different dummy predictor that outputs the mean of the training set labels obtains the worst possible auROC and auPR in this scenario (Fig. S2c,d). Overall, our results indicate a strong information leakage through distributional bias with LOOCV, and that the effect is present even when the held-out test set has more than one sample. Furthermore, we demonstrate this effect to be more pronounced when dealing with significant class imbalances.

### Distributional bias leads to lower performance evaluation of common machine learning models

**Figure 2 |:**
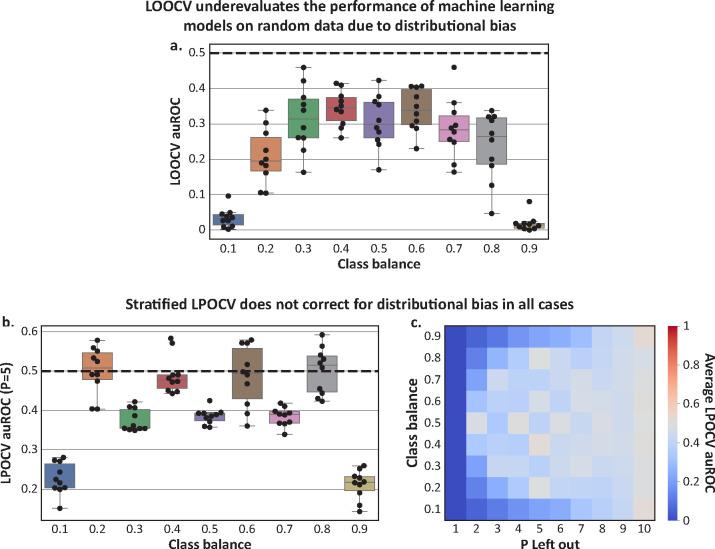
Distributional bias produces results worse than a random guess on random data. All plots pertain to LOOCV and LPOCV analyses of logistic regression models on randomly generated data and labels. The auROC in this setting should be 0.5 in any fair evaluation. **a**, Boxplots of auROCs for a standard LOOCV implementation across different underlying class balances. Resulting auROCs are consistently less than 0.5 (aggregated *p*<0.001 via a single 1-sample t-test). **b**, Boxplots of auROCs on stratified leave-five-out cross-validation across different class balances. When the class balances can be precisely captured with five samples (e.g., class balance of 0.2), the distribution of resulting auROCs has a mean which is not significantly different from 0.5. Otherwise, under-evaluation of performance is evident (e.g., for class balance of 0.1). **c**, Heatmap of average auROCs on stratified LPOCV for Ps ranging from 1 to 10 and for different class balances. Results demonstrate that the distributional bias effect, observed as auROCs below 0.5, is smaller the closer the stratification enabled by P and the class balance is to optimal stratification. Box, IQR; line, median; whiskers, nearest point to 1.5*IQR.

After observing how distributional bias can be used by an adversarial dummy model specifically built to take advantage of this phenomenon, we sought to investigate its impact on the performance evaluation of standard machine learning models. We therefore generated similar simulations as above, with all features drawn i.i.d from uniform distributions ∈[0,1]. Because, by construction, all features and labels are random, any fair assessment of a machine learning model on this data should yield an average auROC of 0.5. However, when assessing the performance of an L2-regularized logistic regression model, a random forest model, and a K-Nearest Neighbor model (K=5) in LOOCV, we instead found auROCs significantly lower than 0.5 across all class balances (one-sample t-test *p* < 0.01 for all vs. 0.5, Figs. 2a, S3). Distributional bias manifests in this way because the models we used tend to predict values close to the class balance, a phenomenon known as regression to the mean (Fig. S4). The effect of distributional bias was more pronounced on logistic regression models (mean±std auROC of 0.23±0.13 across all simulations; Fig. 2a) than on random forests (0.48±0.08; Fig. S3a) and 5-Nearest Neighbor (0.48±0.04; Fig. S3b), in line with stronger regression to the mean observed for the former (Fig. S4). Overall, our results demonstrate that distributional bias tends to decrease the performance of common machine learning models when evaluated using LOOCV.

### Exact stratification corrects distributional bias

As distributional bias results from minor shifts in the class balance of the training set, it would not be present in any evaluation approach that ensures an identical class balance between the training and test sets. To demonstrate this, we first implemented a stratified LPOCV approach, in which we made best effort, given P and the class balance, to maintain an identical class balance between the training and test sets across all folds. When exact stratification was possible, this approach completely resolved distributional bias and led to a fair performance evaluation (Fig. 2c). For example, a leave-2-out CV with a class balance of 50% had a mean±std auROC of 0.50±0.05, and a leave-5-out scheme had auROCs of 0.50±0.06 for 20%, 40%, 60%, and 80% (aggregated; p=0.59 via 1-sample t-test vs. 0.5; Fig. 2b). However, when the class balance cannot be precisely stratified due to a particular combination of P, class balance, and dataset size (e.g., leave-2-out with a class balance of 10%), small shifts in label means will still occur, leading to an under-evaluation of performance. For example, in the leave-5-out scheme and class balances of 10%, 30%, 50%, 70%, and 90%, we observe that all auROCs were below 0.45 (Fig. 2b). These results demonstrate that stratification can correct for distributional bias in LPOCV only when the choice of P facilitates exact stratification.

### A rebalanced leave-one-out CV for distributional bias correction

**Figure 3 |:**
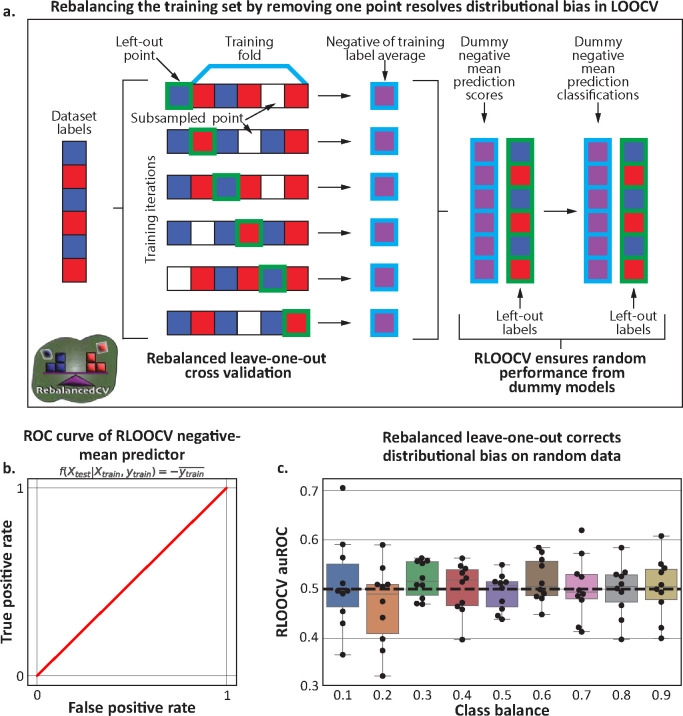
Rebalancing training data through subsampling avoids distributional bias. **a**, An illustration of our proposed rebalanced LOOCV (RLOOCV). For each test point (or fold), we remove from the training set a point with the opposite label such that the training set’s label mean is constant across all folds. This can be accomplished by randomly removing a single training point with a label opposite that of the test point. **b,** ROC curve of the negative-mean predictor (similar to Fig. 1b) evaluated via RLOOCV, which resulted in an auROC of 0.50 (the expected result for an evaluation of a dummy predictor). **c,** Boxplots (Box, IQR; line, median; whiskers, nearest point to 1.5*IQR) of auROCs of a logistic regression model trained on randomly generated data, similar to Fig. 2a, but evaluated with RLOOCV. Resulting auROCs are not consistently higher or lower than the expected 0.5 (*p*=0.84 via a single aggregated 1-sample t-test).

As we demonstrated that stratification cannot always address the effects of distributional bias, we developed a generalizable approach to correct it. We propose a Rebalanced Leave-One-Out Cross Validation (‘RLOOCV’), in which we subsample the training dataset to remove a randomly selected sample of the opposite label as each held-out test sample (Fig. 3a), which ensures that all training sets have the same class balance (even if that balance is slightly different than the class balance across the entire dataset). This approach can be generalized to LPOCV (Supplementary Note 1). We note that an alternative approach could up-sample the held-out class to achieve a similar balance, avoiding loss of samples for training. However, up-sampling and data augmentation involve domain specific knowledge and assumptions^17–19^ and are less generalizable.

To test RLOOCV, we first checked how it evaluates the dummy negative-mean predictor on random data. We found that as RLOOCV maintains a constant training set label mean across all folds, and therefore resolves any potential distributional shifts, the auROC of this adversarial predictor was 0.5 across all class balances (Fig. 3b). Of note, this is different from a stratified leave-2-out CV, which can only address a class balance of 0.5 (Fig. 2c). We observed a similar effect when using RLOOCV to evaluate a logistic regression model on random simulated data across different class balances. While LOOCV evaluated the same model with auROCs consistently lower than 0.5 (Fig. 2a), auROCs evaluated with RLOOCV are not significantly different from 0.5 (*p* = 0.84 via a single aggregated 1-sample t-test, Fig. 3c), as expected for random data. Overall, our synthetic simulations demonstrate that the impact of distributional bias can be corrected by strategic subsampling within a training set, which does not pose any risk of information leakage or of any erroneous inflation of predictive results.

### Rebalancing the training set slightly improves published models evaluated with LOOCV

**Figure 4 |:**
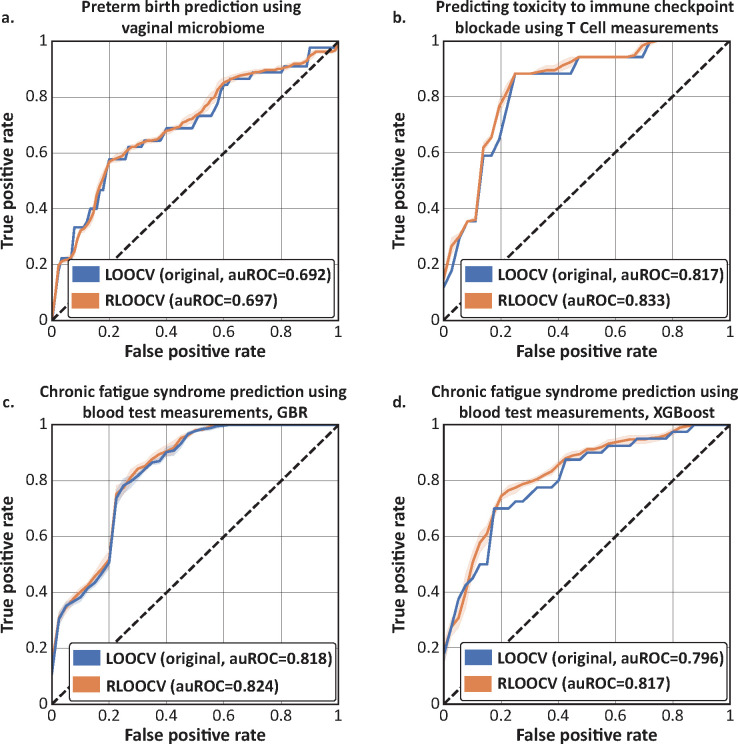
Correcting distributional bias with RLOOCV improves performance evaluation of published predictive models. ROC curves comparing the performance of published models evaluated with LOOCV with the same models evaluated using our rebalancing approach (RLOOCV) over 10 bootstrap runs. Tasks include predicting preterm birth from vaginal microbiome samples using logistic regression^20,21^ (**a**); predicting complications from immune checkpoint blockade therapy using T cell measurements^22^, also using logistic regression (**b**); and predicting chronic fatigue syndrome from standard blood test measurements^22,23^ using gradient boosted regression (**c**) and XGBoost (**d**). Across all cases, we observed a small but consistent improvement from RLOOCV (Fisher’s multiple comparison of DeLong tests *p*=0.015 across all four evaluations). Shaded areas represent 95% confidence intervals.

After evaluating RLOOCV on simulated data, we next sought to investigate how its use would affect published machine learning models that were originally evaluated using LOOCV. To this end, we selected a few cases with available processed data and either code or clear methods. First, we analyzed classifiers of preterm birth from vaginal microbiome samples^20^, using processed data published elsewhere^21^. Using nested 5-fold CV for tuning and evaluation of logistic regression models, we found that while using LOOCV yielded a median auROC of 0.692 (median of 10 bootstrap runs), the median auROC increased to 0.697 with RLOOCV (Fig. 4a). Next, we evaluated prediction of immune-related adverse events (irAEs) in melanoma patients undergoing immune checkpoint blockade using bulk T cell receptor diversity and activated CD4 memory T cell abundances^22^. While repeating the original analysis obtained a median auROC of 0.817 using logistic regression evaluated using LOOCV, we again found a slight increase to a median auROC of 0.833 using RLOOCV (Fig. 4b).

### Rebalancing to account for distributional bias during leave-one-out cross-validation slightly improves results from machine learning analyses

Next, we sought to examine RLOOCV evaluation of more expressive models, such as gradient boosted regression^24,25^. We therefore reproduced an analysis which classified chronic fatigue syndrome from 34 standard blood test measurements^23^. The original analysis trained a gradient boosted regression model, which, when replicated and evaluated with LOOCV, had a median auROC of 0.818. Using RLOOCV, this evaluation increased to 0.824 (Fig. 4c). The original analysis also evaluated XGBoost models, which yielded a median auROC of 0.796. Here we saw a slightly larger improvement with RLOOCV, to a median auROC of 0.817 (Fig. 4d). Altogether, across four different analyses with diverse data types, we observe consistent improvements using RLOOCV implementation (Fisher’s multiple comparison of DeLong^26^ tests *p*=0.015 across all four evaluations). We therefore propose RLOOCV as a low-risk alternative to LOOCV that successfully addresses distributional bias in predictive settings.

### Distributional bias affects hyperparameter optimization

We demonstrated consistent improvements in performance evaluation obtained by correcting distributional biases. While these improvements are small, it could be argued that they are also technical, in that they do not stem from improved predictive capacity of the model itself and therefore would not have a substantial impact on downstream implementations. However, rank-based evaluation metrics are often used not just for performance evaluation, but also for hyperparameter optimization and model selection. To evaluate the potential effects of distributional bias on this optimization, we next focused on regularization, which is often key to obtaining robust model performance.

**Figure 5 |:**
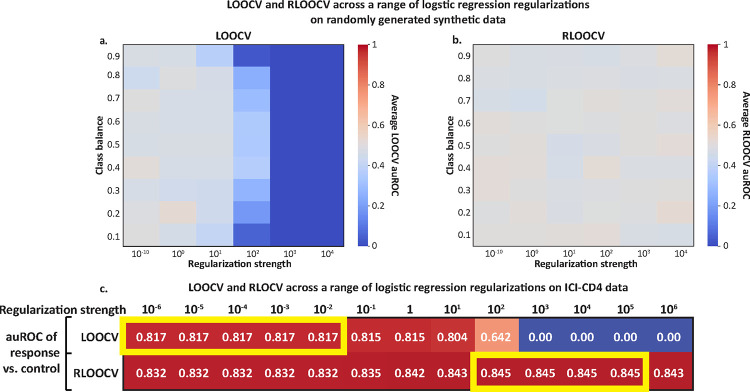
Distributional bias and LOOCV favor weaker regularization. **a,b,** Heatmaps pertain to analyses of logistic regression models on randomly generated data and labels, where the auROC should be 0.5 in any fair evaluation. **a**, Average auROCs evaluated with LOOCV across varying regularization strength and class balances, which are consistently less than 0.5 (*p*<0.001 via 1-sample t-test across all values). **b,** Same heatmap as in (**a**), but with RLOOCV. Resulting auROCs are not consistently higher or lower than 0.5. **c,** Heatmap showing the auROC obtained by logistic regression models classifying patients who experienced complications from immune checkpoint blockade therapy using T cell measurements (Methods). Different rows correspond to evaluation using LOOCV and RLOOCV, while different columns correspond to different regularization strength. The optimal performance in each setup was obtained by RLOOCV. Additionally, the optimal performance was obtained with weaker regularization when evaluated with LOOCV than with RLOOCV, suggesting that distributional bias can cause models tuned via LOOCV to be less regularized.

We first examined our simulations with random data (Fig. 2). Comparing logistic regression models with different regularization strengths using LOOCV and different class balances, we found a stronger impact of distributional bias on models with higher regularization, with auROC=0 in all of the high regularization settings, compared to a mean±std auROC of 0.48±0.07 at the lowest regularization (Fig. 5a). This is because less expressive models with heavier regularization are more likely to predict values close to the label mean, and therefore are more likely to see decreased results due to this distributional shift. Switching to RLOOCV, however, removes the effects of distributional bias. Using the same model and regularization parameters, we observed mean±std auROCs of 0.50±0.06 (*p* = 0.09 via 1-sample t-test vs 0.5 across all models), across a wide range of regularization strength (Fig. 5b).

Next, we hypothesized that as distributional bias has greater performance reduction on models with higher regularization (Fig. 5a,b), it would result in selection of weaker regularization parameters in hyperparameter optimization. To evaluate this hypothesis, we considered the dataset we analyzed in Fig. 4b, in which irAEs were predicted from circulating T-cells characteristics^22^, and evaluated a range of regularization strengths for logistic regression models. We found that auROCs evaluated with RLOOCV were significantly higher than those evaluated with LOOCV across all settings (Wilcoxon signed-rank p=0.0012), with the best auROCs obtained with RLOOCV (auROC of 0.845 compared to 0.817 with LOOCV; Fig. 5c). Interestingly, the regularization level that yielded the best auROCs was different between LOOCV and RLOOCV, with distributional bias causing the best predictor selected with LOOCV to be less regularized than the best predictor selected with RLOOCV (optimal regularization strength of 10^−6^-10^−2^ vs. 10^2^-10^5^, respectively, Fig. 5c). Consistent with our simulations, the impact of distributional bias is particularly evident in the high-regularization settings, in which the distributional bias carries a relatively higher importance. In those scenarios, which could often be relevant for inference, we see LOOCV-evaluated auROCs of 0, while the auROCs evaluated with RLOOCV can have optimal performance (Fig. 5c). Overall, these results demonstrate that distributional bias would cause hyperparameter optimization performed using non-balanced cross-validation to select suboptimal regularization parameters.

## Discussion

Distributional bias emerges when there is a shift across the mean of the labels of different training sets, and the rankings of predictions across all folds are considered jointly. We show that with an adversarial model, such as a dummy predictor that always predicts the negative of the average training set label, this would result in information leakage and a perfect auROC and auPR, regardless of the underlying data. In practice, however, we show that since many machine learning models tend to regress to the mean, distributional bias would actually cause an under-evaluation of their predictive performance under LPOCV, especially when P is small or when the class ratio is imbalanced. We demonstrate this effect using published test cases of LOOCV evaluation of machine learning models, and show that this phenomenon may also lead to a suboptimal selection of hyperparameters. To address this, we propose RLOOCV, a generalizable solution to maintain class balances across all training sets, by subsampling the training sets to ensure consistent label distributions.

While we demonstrate that distributional bias causes a consistent under-evaluation of performance using LOOCV, we note that the strength of this effect varies across models. In particular, this effect is strongest when models are highly regularized, in which case they are more likely to produce class probabilities that are closer to the training set label average. In hyperparameter optimization, the distributional bias we observe in LOOCV would push towards selection of weaker regularization, which might then negatively affect subsequent generalization and performance on these models on additional datasets and limit interpretation and explainability of optimized models.

While we present RLOOCV, in which an additional training sample is removed along with the test sample as a generalizable solution to this problem, we note a few other possible corrections that may, under certain conditions, address distributional bias: 1) stratified LPOCV, so long that the class balance can be strictly maintained in all train and test folds; 2) an “up-balanced” LPOCV, in which a sample from the same class is generated for each sample that is held out as a test set; 3) a modification of RLOOCV, in which for every left-out fold, a separate model is trained for every possible subsampled training dataset that maintains the desired class balance; and 4) similarly to (3) one could perform bagging^27^ to train a collection of different models across different random subsets of the training set, with each individual bag from every training fold containing the same class balance. Lastly, while we note that a post-hoc normalization of a model’s predictions with respect to either the average training set labels or the model’s predictions on the training set seems like an intuitive solution, we found scenarios in which this approach overestimated performance evaluation, such as K-Nearest Neighbors models with small K (Supplementary Note 2). Therefore, we do not recommend post-hoc normalization as a valid solution to distributional bias.

## Methods

### Synthetic simulations

For each simulation, we randomly generated a set of N binary labels $y_{i}\in \{0,\;1\}$, for i∈[1,2,…,N], with class balances ranging from 0.1 to 0.9 in increments of 0.1. Data was generated by drawing for every sample a collection of 20 i.i.d features from a uniform distribution on [0,1]. The “negative-mean predictor” was defined as ${\overset{ˆ}{y}}_{i}=-\frac{\sum_{j\neq i}\;y_{j}}{N-1}$, where ${\overset{ˆ}{y}}_{i}$ is the prediction for each held-out sample $i,\;y_{j}\in \{0,\;1\}$ are the training labels for each sample j, with a total of N samples across the entire dataset. For each simulation setting (e.g., each cell in Fig. 2c), we generated 100 random datasets of 250 or more points, where in certain scenarios we used more than 250 so that the P-left-out would divide evenly into the dataset size. In the case of random forest simulations (Fig. S3a), we simulated only 100 points per dataset to minimize computational runtime.

### Benchmarking of predictive analyses

We considered datasets which had previously been analyzed using LOOCV, had publicly available processed data, and had public code or clear methods. To this end, we considered datasets from Vogl et al^23^, Lozano et al.^22^, and Fettweis et al.^20^. The first two processed datasets and corresponding code were obtained from the original publications, while the third processed dataset was obtained from a different study with publicly available materials^21^, which we processed as described, using a pseudocount of 10^−6^ and centered log ratio transform, followed by LOOCV and RLOOCV comparisons of tuned logistic regressors. In all cases, we show results from 10 bootstrap runs (Fig. 4). For the analysis of Lozano et al., we also demonstrate performance results across thirteen regularization strengths (Fig. 5c)

## Supplementary Material

Supplement 1

## Data Availability

All datasets used in this study are publicly available. Datasets used for simulations are generated by the scripts in our analysis repository (https://github.com/korem-lab/LPOCV-bias-analysis). The chronic fatigue syndrome, preterm birth, and immune checkpoint inhibitor datasets are available from the original publications^21–23^.
