# Supplementary material for: Distributional bias compromises leave-one-out cross-validation
Source: ArXiv. 2024 Jun 3:arXiv:2406.01652v1. Preprint. [Version 1] (PMC11177965)
Supplement: Supplementary file 1 [file NIHPP2406.01652v1-supplement-1.pdf]

Supplementary Notes

Supplementary Note 1 | Generalizing RLOOCV to RLPOCV and RKFold

Consider a dataset consisting of T positive samples, F negative samples, and a desired P left-out schema. The simple approach to rebalance LPOCV per held out set is to randomly remove P cases from the training set with labels opposite those of the held-out set, such that every model is trained using exactly $T + F - 2P$ observations, consisting of $T - P$ positive and $F - P$ negative samples. However, acknowledging that LPOCV would be considered in scenarios that are already data-sparse, we show that using stratification for the held out test set, it is possible to develop a stratified RKFold schema that

removes fewer samples from the training set, which can be especially important in a highly imbalanced dataset with few observations from one class. We consider the following:

- $N = T + F//P$, the total number of P-sized held-out groups.
- $T_c = T//N$, the total number of positive class observations that are guaranteed to be present in every left-out P group.
- $F_c = F//N$, the total number of negative class observations that are guaranteed to be present in every left-out P group.

Therefore, every held-out group consists of:

1. T_c positive samples.
2. F_c negative samples.
3. $P - (T_c + F_c)$ remaining samples, which will cause label averages to vary across training folds, leading to distributional bias.

Because components (1) and (2) are constant across all training folds, there is no need to subsample training datasets to match those held out samples. The only subsampling needed to ensure consistent class balances across all training folds is to remove samples opposite each case in (3). Therefore, when performing stratified RLPOCV, each training fold should use exactly:

- $T - P + F_c$ positive samples
- $F - P + T_c$ negative samples

To demonstrate a few examples, under this RLPOCV implementation, for a dataset with T=50, F=50, and P=5, each model will be trained using exactly 47 T and 47 F samples. For a different dataset with T=10, F=1000, and P=2, each model will be trained using exactly 9T and 998 F samples. We note that in the case of P=1, RLPOCV is identical to the RLOOCV implementation above.

Supplementary Note 2 | Post-hoc standardization addresses distributional bias but may lead to performance inflation.

An intuitive approach for correcting distributional bias is to normalize (scale, z-score, etc.) a model's predictions on the held-out points with respect to the same model's predictions on its training set. We describe this approach here as a cautionary tale, as it is unsuitable in certain settings. In some of our simulations, such as those analyzed with logistic regression, this approach performs exactly as intended, resulting in auROCs close to the expected 0.5 (p = 0.14 via 1-sample t-test; **Fig. S5a**). However, in cases in which a model does not have the flexibility to precisely predict a class balance, for instance only being capable of predicting 0 or 1, then any normalization with respect to training label averages will create perfect rankings within all samples initially predicted at the same value. The simplest example of this is the K-Nearest Neighbor model with K=1: When running this predictor through our same simulation framework but including a post-hoc z-score standardization to the training predictions, we observe auROCs larger than the intended 0.5 in every case (p < 0.001 via 1-sample t-test; **Fig. S5b**), and with auROCs greater than 0.8 at extreme class imbalances. Although there are cases in which this normalization strategy is not problematic, we see no generalizable and concrete methods to

ensure that elements of the more problematic models are not present in a given situation. Therefore, we conclude that there is always some risk of over-evaluation of machine learning performance when using a post-hoc normalization, and we recommend to avoid using it as a solution to distributional bias. Instead, RLOOCV solves this challenge without introducing any risk of overinflating results.

Supplementary Figures

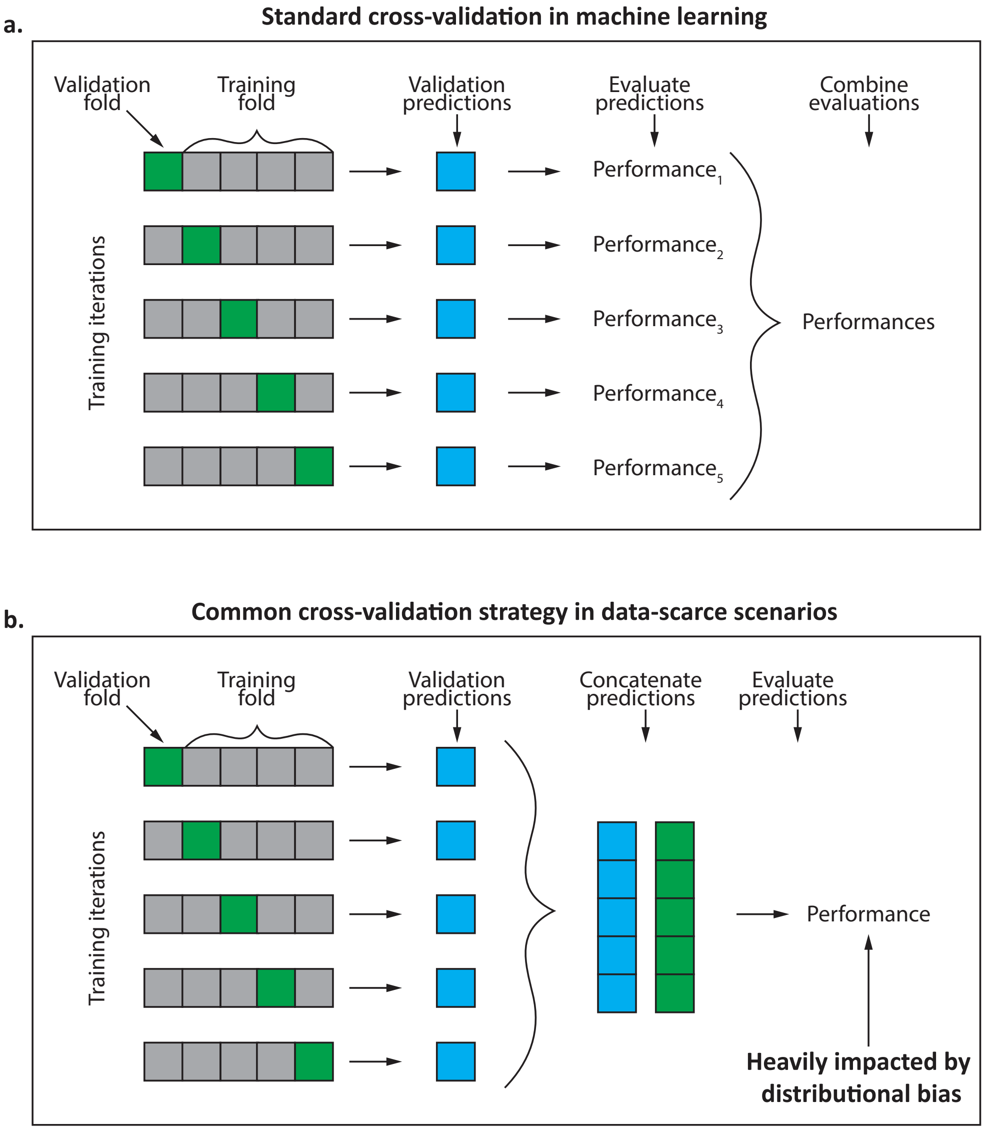

Figure S1 | Comparison of cross-validation strategies. a, Description of standard cross-validation approaches in machine learning, in which performance evaluation is performed separately for each held-out test set. **b,** A commonly used cross-validation strategy in data-scarce scenarios, in which the evaluations across all folds is performed once on the concatenation of all predictions. The evaluation approach in (**b**) can be heavily impacted by distributional bias. Figure partially adapted from ref. [6].

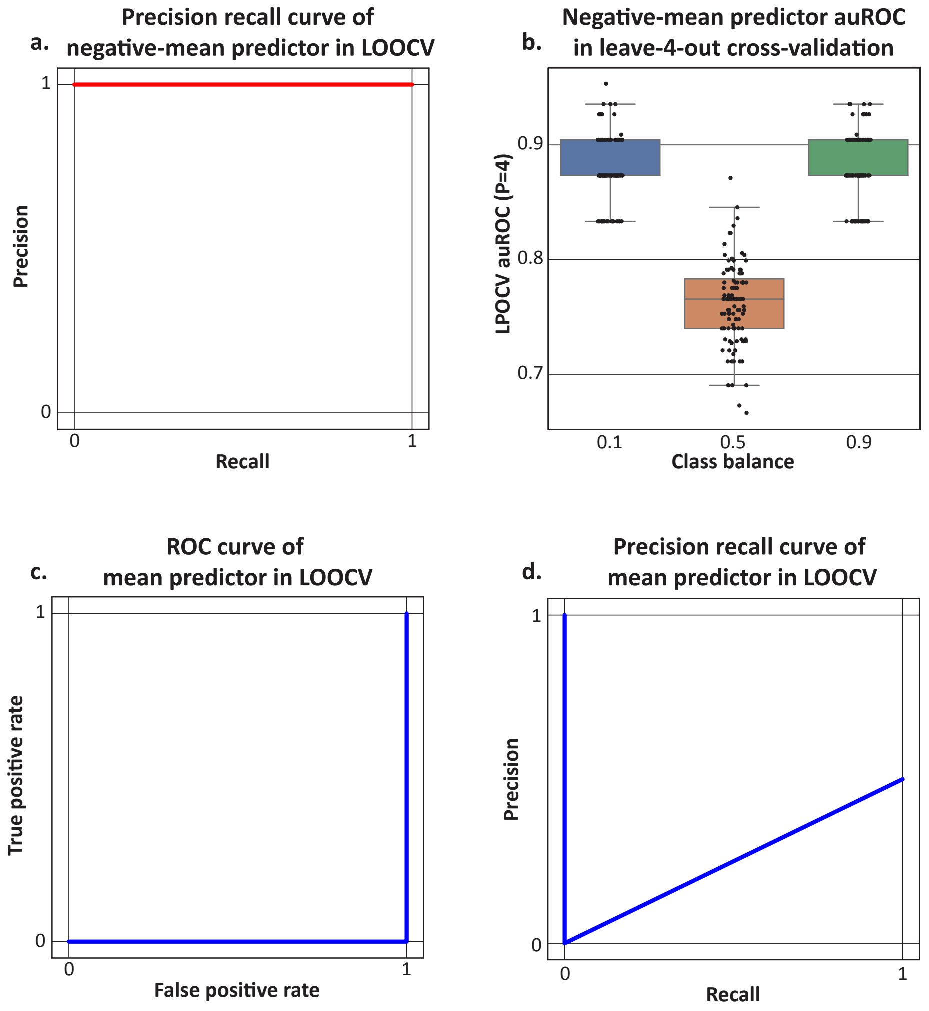

Figure S2 | Impact of distributional bias across dummy models and evaluation metrics. a, Results from the same model as in **Fig. 1b**, shown in a precision-recall curve, with an area under the curve of 1. **b,** Box and swarm plot demonstrating results from the same set of models as in **Fig. 1c**, for P=4 and class balances of 10%, 50%, and 90%. Box, IQR; line, median; whiskers, nearest point to 1.5*IQR. **c,d** ROC and PR curves similar to **Fig. 1b** and **Fig. S2a**, but for a predictor that outputs the label mean of the training set. Under LOOCV evaluation, for a class balance of 0.5, auROC=0 and auPR=0.25.

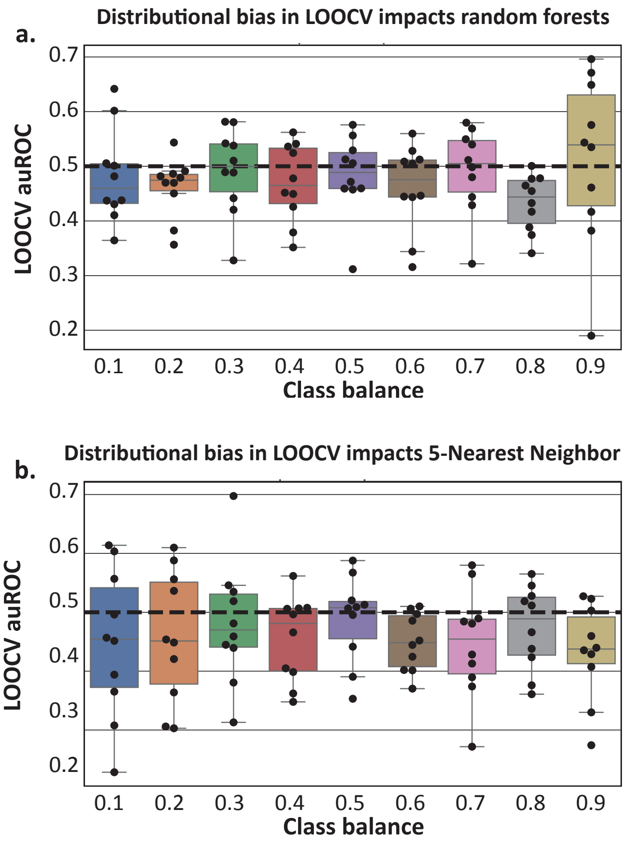

Figure S3 | Distributional bias impacts random forest and 5-Nearest Neighbor models. a,b, Boxplots demonstrating similar results as in **Fig. 2a**, but for random forest models (a), and K-Nearest Neighbors with K=5 (b). The resulting auROCs are consistently lower than 0.5 (p=0.009 via 1-sample t-test vs. 0.5 for random forest, p=2.9×10^{-5} for K-Nearest Neighbors). Box, IQR; line, median; whiskers, nearest point to 1.5*IQR.

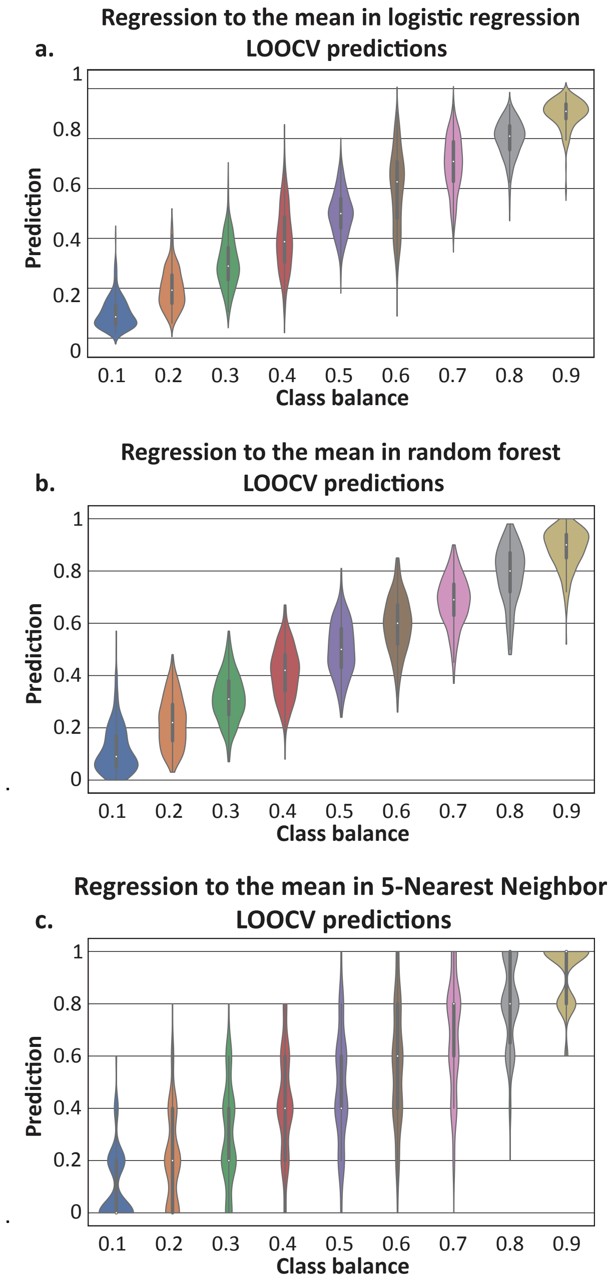

Figure S4 | Regression to the mean in common machine learning models. a-c, Violin plots showing predictions of logistic regression (**a**), random forest (**b**), and 5-Nearest Neighbors (**c**) models on randomly generated datasets in LOOCV, across different underlying class balances. All models used default scikit-learn parameters.

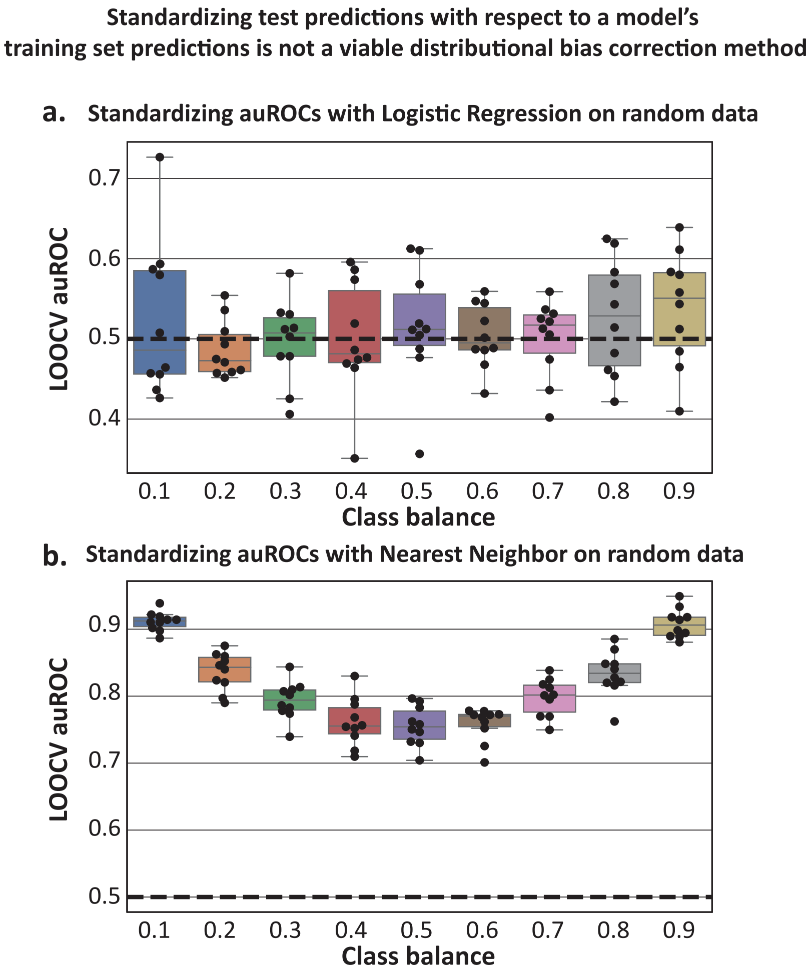

Figure S5 | Post-hoc prediction standardization may lead to over-evaluation of performance. Boxplots demonstrating a similar analysis as in **Fig. 2a**, but all test predictions are standardized to the model's predictions on the training set via z-scoring. **a,** Logistic regression evaluation standardized in this way produces results similar to the expected random guess, with an auROC close to 0.5 ($p = 0.14$ via a single 1-sample t-test of the aggregated results). **b,** 1-Nearest Neighbor models in the same standardized evaluation framework yield results larger than 0.5 ($p < 0.001$ via a single 1-sample t-test of the aggregated results). Box, IQR; line, median; whiskers, nearest point to 1.5*IQR.
